# Clinical Significance of C-Reactive Protein to Albumin Ratio in Patients with Hepatocellular Carcinoma: A Meta-Analysis

**DOI:** 10.1155/2020/4867974

**Published:** 2020-09-02

**Authors:** Nanping Lin, Jingrong Li, Qiao Ke, Lei Wang, Yingping Cao, Jingfeng Liu

**Affiliations:** ^1^Department of Hepatopancreatobiliary Surgery, Mengchao Hepatobiliary Hospital of Fujian Medical University, Fuzhou, Fujian, China; ^2^The First Affiliated Hospital of Fujian Medical University, Fuzhou, Fujian, China; ^3^Department of laboratory, Fujian Medical University Union Hospital, Fuzhou, Fujian, China; ^4^Department of Radiation Oncology, Mengchao Hepatobiliary Hospital of Fujian Medical University, Fuzhou, Fujian, China

## Abstract

**Aim:**

To evaluate the prognostic significance of C-reactive protein to albumin ratio (CAR) for clinical outcomes in hepatocellular carcinoma (HCC) patients. *Material and Methods.* Eligible studies were searched by PubMed, MedLine, the Cochrane Library, from January 1, 2000, to June 30, 2019, investigating the prognostic value of CAR in patients with HCC. Primary endpoint was OS. Hazard ratio (HR) with 95% confidence interval (CI) was used to determine the effect size.

**Results:**

7 records including 2208 patients published since 2014 were enrolled into our meta-analysis. Clinicopathological characteristics were also correlated with the level of CAR. The pooled HR for the OS rate between low and high CAR groups was 2.13 (95% CI 1.70~2.68, *P* < 0.00001) using a random model, but sensitivity analysis showed that the pooled HR for the OS rates did not change substantially after removal of any included study. As for patients receiving surgery, the pooled HR for the OS rate between low and high CAR groups was 2.04 (95% CI 1.59~2.61, *P* < 0.00001). Subgroup analysis showed that CAR could be a prognostic biomarker for HCC patients regardless of regions (China, HR = 1.75, 95% CI 1.51~2.02; Japan, HR = 3.36, 95% CI 2.07~5.45; Korea, HR = 2.26, 95% CI 1.47~4.47; respectively), the cut-off value (<0.1, HR = 2.84, 95% CI 1.90~4.24; >0.1, HR = 1.99, 95% CI 1.52~2.61; respectively), and sample size (<200, HR = 2.85, 95% CI 2.01~4.03; >200, HR = 1.75, 95% CI 1.52~2.02; respectively).

**Conclusion:**

With the current data, we clearly concluded that CAR was closely correlated with prognosis of patients with HCC. Multicenter, prospective randomized trials are warranted to confirm the conclusion.

## 1. Introduction

The incidence of hepatocellular carcinoma (HCC) is increasing stably worldwide, but the prognosis still far from satisfactory [1]. Radical resection is still one of the most efficient strategies to cure HCC, but the incidence of recurrence at 5-year is reported to be as high as 70-80% [2, 3]. Considering 80% of patients have lost the chance of surgery at diagnosis [4], strategies for HCC varied from different stages [5, 6]. Hence, biomarkers served as predictors of prognosis and aid of decision-making are badly needed in clinical.

C-reactive protein to albumin ratio (CAR) has been reported as a powerful prognostic indicator for solid tumors [7–9], and it has been confirmed in colorectal cancer, esophageal cancer, and nasopharyngeal cancer by several newly published meta-analysis [10–12]. Reasons might be as follows: (1) many of the solid tumors are highly associated with inflammation, and C-reactive protein (CRP) is one of the most common kinds of systematic inflammatory index; (2) nutritional status is one of the crucial factors for the long-term prognosis of patients with cancers, which is attracting more and more attentions, and albumin (ALB) level is the simplest marker to evaluate the status of nutrition.

Recently, high CAR has been reported to be correlated with poor prognosis of patients with HCC [13–19], but the results varied from each other. And to the best of our knowledge, there are no meta-analysis and systematic review evaluating the prognostic value of CAR in patients with HCC. Therefore, a meta-analysis was warranted to determine the prognostic significance of CAR for clinical outcomes in HCC patients.

## 2. Material and Method

This study was designed according to PICOS principles and conducted based on the preferred Reporting Items for Systematic Reviews and Meta-Analyses (PRISMA) statement [20].

### 2.1. Literature Search

A comprehensive search on the existing published medical literature was conducted by Jingrong Li and Nanping Lin to investigate the prognostic value of CAR for patients with HCC. English electronic databases such as PubMed, MedLine, and Embase were used to search the literature from January 1, 2000, to June 30, 2019. Key words were as follows: ((“hepatocellular” or “liver” or “hepatic”) AND “tumour” or “tummor” or “cancer” or “carcinoma” or “neoplasm”) OR (“HCC” or “LC”)) AND (“C-reactive protein” or “albumin” or “CAR”). Any potentially eligible studies were then identified manually through the references of the included studies, reviews, letters, and comments [21].

### 2.2. Selection Criteria

Inclusion criteria: (i) patients with clinic or pathological confirmed HCC; (ii) pretreatment CRP and albumin was determined; (iii) clinical outcomes including overall survival (OS), disease-free survival (DFS).

Exclusion criteria: (i) patients including benign disease or other tumors; (ii) only CRP or albumin was detected before any treatment; (iii) patients showed clinical evidence of inflammatory conditions rather than hepatitis were also excluded; (iv) data on the clinical outcomes was not available; (v) in vivo studies; (vi) conference abstracts, reviews, letters, and comments.

### 2.3. Endpoints

Primary endpoint was OS. Secondary endpoints were DFS.

### 2.4. Data Extraction

Data such as the author's information, year of publication, patient's basic characteristic, cut-off value, follow-up time, and outcomes were extracted and assessed by Jingrong Li and Nanping Lin with predefined forms. The hazard ratios (HRs) of OS were extracted directedly from the original data or extracted from the Kaplan-Meier curves according to the methods described in detail by Tierney et al. and Parmar et al. In case of disagreement, a third investigator, Qiao Ke, was intervened to reach a conclusion [21].

### 2.5. Quality Assessment

The quality of nonrandomized studies was assessed by the modified Newcastle-Ottawa Scale (NOS), and more than 6 stars were defined as high quality, 4~6 stars as medium quality, and less than 4 stars as low quality.

### 2.6. Statistical Analysis

The meta-analysis was registered at http://www.crd.york.ac.uk/PROSPERO/ (Review registry 143152) and was performed using RevMan Version 5.3. The pooled HRs for OS between high and low levels of CAR were evaluated with 95% Cis. The effects mode that was used depended on the heterogeneity, which was assessed by the *χ*^2^ test and *I*^2^ statistics; *P* < 0.10 or *I*^2^ >50% were considered as significant heterogeneity, and random-effected was chosen. When the hypothesis of homogeneity was rejected, the fixed-effects model was used to estimate the case with homogeneity. Sensitivity analysis was conducted as follows: one study at a time was removed, and the remained were reanalyzed to determine whether the results could be affected significantly by single study. Begg's and Egger's tests were used to evaluate publication bias using Stata 14. Trim and filling method was used to evaluate the stability of the result if *P* < 0.05 [21].

## 3. Results

### 3.1. Base Characteristic of the Included Studies

**Figure 1:**
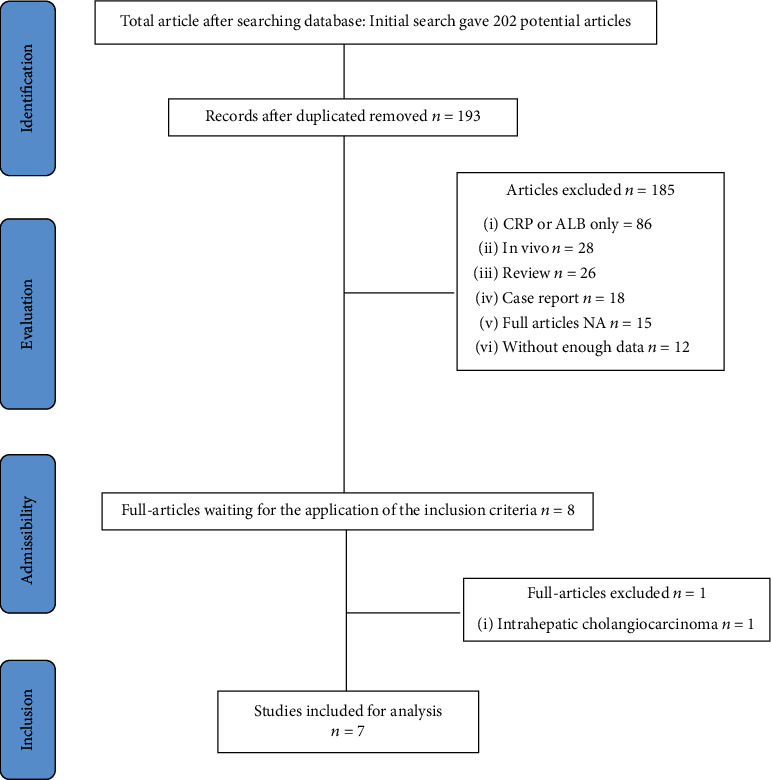
PRISMA flow diagram showing selection of articles for meta-analysis.

Totally, 202 records were identified by Jingrong Li and Nanping Lin. 9 records were excluded for duplication by NoteExpress 3.1, and then, 186 records were excluded after browsing titles and abstracts. Hence, 7 records [13–19] including 202 patients published since 2014 were enrolled into our meta-analysis. The search results and details were shown in Figure 1.

The characteristics and baseline demographic data of the patients in each research were listed in Table 1. Of note, ALB to CRP ratio was reported in one study [19] and transferred it into CAR accordingly, which was confirmed repeatedly by Jingrong Li, Nanping Lin, and Qiao Ke. All studies were scored above 6 by NOS.

Clinicopathological characteristics were also correlated with the level of CAR. As shown in Table 2, the mean level of AFP in the high group was higher than that in the low group (28-38.5 ng/ml vs 8-28 ng/ml), and the tumor in the high group was bigger than that in the low group (1.0-20.0 cm vs 0.7-5.2 cm). As shown in Table 3, high CAR was found to be with multiply tumors and advanced TNM stage.

### 3.2. Primary Endpoint

**Figure 2:**
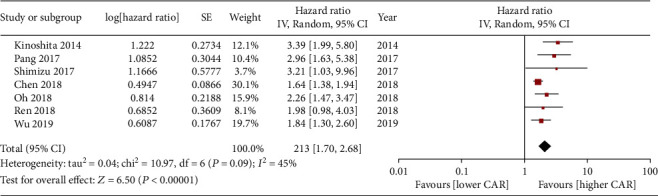
Forest plot of the pooled HR for the OS rates comparing between low and high CAR groups.

The OS rates comparing between low and high CAR groups were evaluated in 7 included studies [13–19]. Heterogeneities were observed (*I*^2^ = 45%, *P* = 0.09), and using a random model the pooled HR for the OS rate between low and high CAR groups was 2.13 (95% CI 1.70~2.68, *P* < 0.00001, Figure 2(a)).

Heterogeneities disappeared after removing Chen's study [17] (*I*^2^ = 0, *P* = 0.44), and then, the pooled HR for the OS rate between low and high CAR groups was 2.31 (95% CI 1.87~2.84, *P* < 0.00001, Figure 2(b)) using a fixed model, which indicated that the results were considerably reliable.

### 3.3. Subgroup Analysis of Hepatectomy for HCC

**Figure 3:**
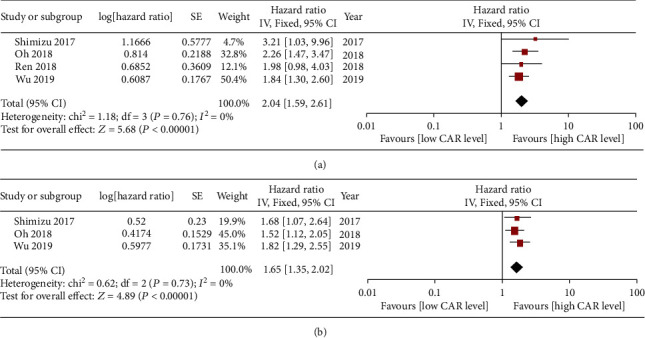
Subgroup analysis of hepatectomy for HCC. (a) Forest plot of the pooled HR for the OS rates comparing between low and high CAR groups. (b) Forest plot of the pooled HR for the DFS rates comparing between low and high CAR groups.

The OS rates of HCC patients receiving hepatectomy comparing between low and high CAR groups were evaluated in four included studies [15, 16, 18, 19]. Significant heterogeneity was not observed (*I*^2^ = 0, *P* = 0.76), and using a fixed model. The pooled HR for the OS rate between low and high CAR groups was 2.04 (95% CI 1.59~2.61, *P* < 0.00001, Figure 3(a)).

The DFS rates of HCC patients receiving hepatectomy comparing between low and high CAR groups were evaluated in three included studies [15, 16, 19]. Significant heterogeneity was not observed (*I*^2^ = 0, *P* = 0.73); then, fixed model was selected. The pooled HR for the OS rate between low and high CAR groups was 1.65 (95% CI 1.35~2.02, *P* < 0.00001, Figure 3(b)).

### 3.4. Subgroup Analysis of the Correlation between CAR and OS

As summarized in Table 4, subgroup analysis of OS stratified by regions, the cut-off value for CAR, and the sample size were conducted. Results showed that CAR could be a prognostic biomarker for HCC patients regardless of regions (China, HR = 1.75, 95% CI 1.51~2.02; Japan, HR = 3.36, 95% CI 2.07~5.45; Korea, HR = 2.26, 95% CI 1.47~4.47; respectively), the cut-off value (<0.1, HR = 2.84, 95% CI 1.90~4.24; >0.1, HR = 1.99, 95% CI 1.52~2.61; respectively), and sample size (<200, HR = 2.85, 95% CI 2.01~4.03; >200, HR = 1.75, 95% CI 1.52~2.02; respectively).

### 3.5. Sensitivity Analysis

**Figure 4:**
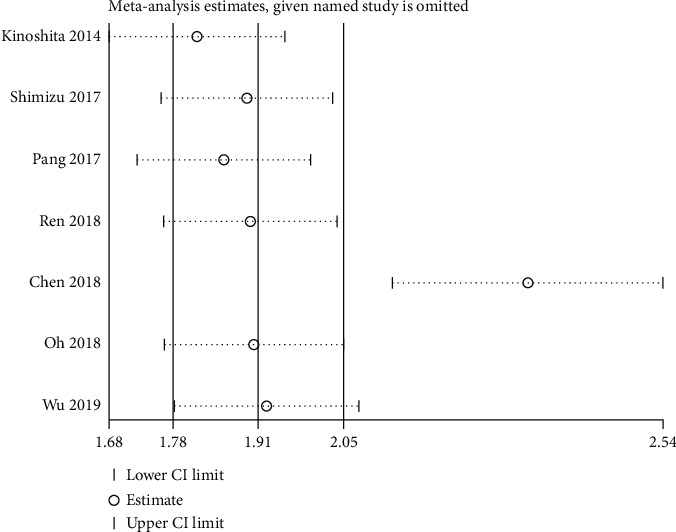
Sensitivity analysis for the pooled OS comparing between low and high CAR groups.

Sensitivity analysis was conducted for the pooled OS comparing between low and high CAR groups. Results showed that the pooled OS rates comparing between low and high CAR groups did not change substantially after removal of any included study (Figure 4), which indicated that the results were considerably reliable.

### 3.6. Publication Bias Analysis

**Figure 5:**
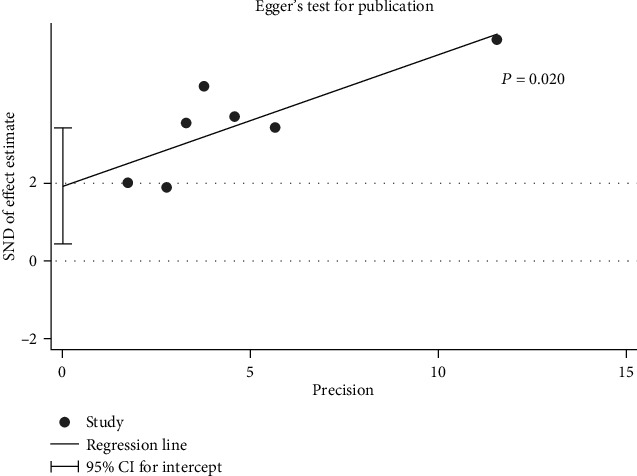
Egger's test for publication bias.

**Figure 6:**
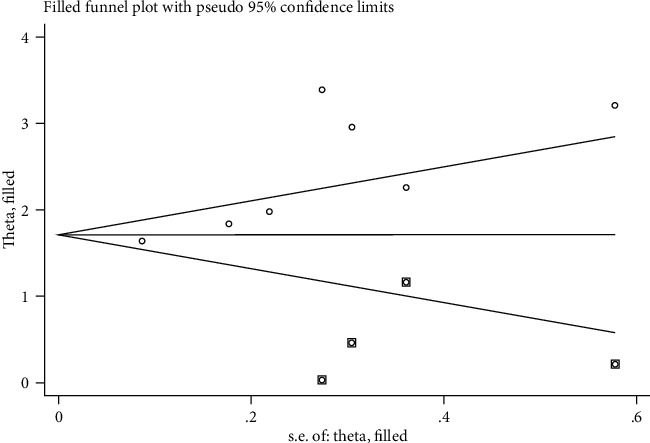
Funnel plot after adjusted by trim and fill method.

The publication bias analysis was conducted for the pooled HR for the OS rates comparing between low and high CAR groups, and results showed that significant publication biases were observed in the Egger's test (*P* = 0.02, Figure 5), but no significant publication biases were observed in OS comparing between high and low level of CAR using the Begg's test (*P* = 0.23). Trim and fill method was conducted to assess the stability of the result. After “trim and fill” analysis, four more studies were enrolled, and the pooled HR for the pooled OS rates comparing between low and high CAR groups was 1.744 (1.221-2.267), which indicated that the unpublished studies would not change the results. Funnel plot after adjusted was shown in Figure 6.

## 4. Discussion

This is the first meta-analysis addressing the prognostic value of CAR in HCC. Seven studies were eligible including 202 patients, and results showed that CAR was not only correlated with clinicopathological characteristics but was also associated with OS of patients with HCC. What's more, the results were confirmed by subgroup analysis and sensitivity analysis. Hence, CAR could be served as a prognostic biomarker for patients with HCC.

As noninvasive and obtained easily in clinic indicators, serum biological markers have always been considered as the ideal biomarkers for the prognosis in tumor [22, 23]. CAR, as a star prognostic biomarker for cancer [24, 25], two of whose elements, CRP and ALB, are derived from blood. Recently, CAR has been confirmed by several meta-analysis in colorectal cancer [12], esophageal cancer [11], and nasopharyngeal cancer [10], except hepatocellular carcinoma. In this meta-analysis, we found that high CAR was associated with poor prognosis for all patients with HCC. As for patients receiving surgery, high CAR was associated with poor OS and DFS, which indicated that CAR could be applied widely.

The roles of CAR go far beyond the prediction of prognosis. AFP is the most common indicator used to screen early HCC [26, 27]; in this study, we found that the level of CAR was closely associated with the concentration of AFP, indicating that CAR could be served as a biomarker for HCC screening. Tumor number [28, 29] and tumor diameter are the two important indexes for HCC staging, and high CAR were confirmed to be associated with more tumor sites and bigger ones, suggesting that CAR could be a predictor for tumor staging.

However, mechanism underlying the prognostic value of CAR in HCC remains unclear [30, 31]. Potential explanations might be as follows: (1) HCC is a classical inflammation-related cancer, which is often progressed from hepatitis-cirrhosis [4, 32], and nonsteroidal anti-inflammatory drugs such as aspirin are confirmed to be able to reduce the risk of morbidity and mortality of HCC [33, 34]; (2) the role of nutrition status is tended to be more and more important in the prognosis of cancer, especially in advanced cancers, and as a direct index of nutrition status, ALB is only synthesized in liver [15, 35, 36]. Hence, in our opinion, the prognostic value of CAR is much bigger in HCC than that in other solid tumors.

There were several limitations in this study. First, all the included studies were retrospective studies, indicating an obvious recalling bias. Second, all the included studies came from Japan, South Korea, and China, indicating an apparent regional bias because the epidemiology differed between the West and East. Third, patients receiving surgery or not were enrolled into four of the included studies, indicating obvious confounding bias. Fourth, the cut-off value of CAR varied from each included study. Fifth, dynamic changes in CAR were considered to be much more meaningful than pretreatment CAR, but only one study [18] on this topic was identified. The last but not the least, publication bias was hard to be avoided, although significant publication bias was not detected after “trim and fill” analysis.

## 5. Conclusion

With the current data, we concluded without questions that CAR was closely correlated with prognosis of patients with HCC and could be applied as a noninvasive prognostic biomarker for HCC in clinic. In future, the cut-off value of CAR should be determined. However, given the restrictions mentioned above, multicenter, larger sample, and prospective randomized trials are warranted to confirm this meta-analysis.

## Figures and Tables

**Table 1:** Basic characteristic of the included article.

Studies	Country	Study year	Tumor type	Cut-off value	Study sample (L/H)	Received therapy	Follow-up time (month)	Prime endpoint	NOS score
Kinoshita 2014	Japan	2005-2012	HCC	0.037	186 (84/102)	Multiply	18 (1-88)	OS	7
Pang 2017	China	2007-2014	HCC	—	139	Surgery	23.1 (0.4-103.2)	OS/RFS	7
Shimizu 2017	Japan	2006-2013	HCC	0.028	239 (84/155)	Surgery	—	OS/RFS	8
Oh 2018	Korea	2004-2013	HCC	0.625	389	Surgery	—	OS/RFS	7
Ren 2018	China	2012-2017	HCC	0.037	187 (95/92)	Multiply	23 (1-60)	OS/TFS	8
Chen 2018	China	2013-2016	HCC	—	659	—	21.6 (1-52.7)	OS	6
Wu 2019	China	2008-2012	HCC	0.185	409 (236/173)	Multiply	Until Jun 30 2016	OS	8

HCC: hepatocellular carcinoma; NOS: Newcastle-Ottawa Scale; “-”: not mentioned.

**Table 2:** Correlations between low and high CAR groups with clinicopathological characteristics unable to conduct with meta-analysis.

Studies factor	Kinosta 2014		Shimizu 2017		Ren 2018	
	Low	High	Low	High	Low	High
AFP (*μ*g/L) (mean and range)	17.7 (2–1,693)	36 (1–280,6)	8 (4–85)	28 (6–337)	28 (19–49)	38.5 (25.3–56.0)
Maximum tumor size (cm)	2.5 (0.7–8.8)	4.3 (1.0–20.0)	2.5 (1.8–3.7)	4.0 (2.3–6.5)	3.5 (2.7–5.2)	6.0 (4.34–9.5)

**Table 3:** Correlations between low and high CAR groups with clinicopathological characteristics conducted with meta-analysis.

Items factors	Included studies	OR (95% CI)	*P* value	*I* ^2^	Analysis model
Liver cirrhosis	2	1.07 (0.72,1.59)	0.75	0%	Fixed
Multiply tumors	3	0.57 (0.69,0.84)	0.005	17%	Fixed
Child grade A	2	0.61 (0.20,1.91)	0.40	85%	Random
MVI	2	0.56 (0.25,1.27)	0.17	63%	Random
TNM I, II, III, and IV	3	0.43 (0.25,0.72)	0.001	46%	Fixed

**Table 4:** Subgroup analysis of the correlation between CAR and OS in different factors.

Subgroups	Included studies	Pooled HR value	95% confidence interval	*P* value	Heterogeneity	
					*I* ^2^	*P* value
Region						
China	4	1.75	1.51, 2.02	<0.001	20	0.29
Japan	2	3.36	2.07, 5.45	<0.001	0	0.93
Korea	1	2.26	1.47, 4.47	<0.001	—	—
Cut-off value						
<0.1	3	2.84	1.90, 4.24	<0.001	0	0.48
>0.1	2	1.99	1.52, 2.61	<0.001	0	0.47
Sample						
<200	3	2.85	2.01, 4.03	<0.001	0	0.49
>200	4	1.75	1.52, 2.02	<0.001	3	0.38

## Data Availability

Answer: Comment: N/A
